# Relationship between Household Shared Meal Frequency and Dietary Intake among Men and Women Aged ≥20 Years: Cross-Sectional Analyses Based on 2018 and 2019 National Health and Nutrition Surveys in Japan

**DOI:** 10.3390/nu16111764

**Published:** 2024-06-04

**Authors:** Xiaoyi Yuan, Mai Matsumoto, Emiko Okada, Kentaro Murakami, Satoshi Sasaki, Hidemi Takimoto

**Affiliations:** 1 Department of Nutritional Epidemiology and Shokuiku, National Institutes of Biomedical Innovation, Health, and Nutrition, 3-17 Senrioka Shinmachi, Settsu-shi 566-0002, Osaka, Japan; 2 Department of Social and Preventive Epidemiology, School of Public Health, University of Tokyo, Bunkyo-ku 113-0033, Tokyo, Japan

**Keywords:** household shared meals, dietary record, adults, Japanese, National Health and Nutrition Survey

## Abstract

This study aimed to examine the relationship between the frequency of household shared meals and the intake of 17 food groups and 21 nutrients. Participants were 3310 men and 3386 women ≥20 years old living in a household of ≥2 members from 2018 and 2019 National Health and Nutrition Surveys in Japan. A one-day household dietary record was used to classify an individual’s shared meal frequency and dietary intake. A shared meal is defined as an eating occasion (i.e., breakfast, lunch, and dinner) where ≥1 food item—other than sugars, fats and oils, beverages, and condiments—was recorded with an assigned approximated shared proportion. The shared meal frequency for each individual was classified into one of four categories: 0, 1, 2, and 3 times/day. Dietary intake was compared across the shared meal categories adjusted for age, occupation, household size, meal skipping, snacking, residential areas, and within-household correlations. Both men and women who had more frequent shared meals showed higher intakes of potatoes, vegetables, mushrooms, and condiments but less confectioneries and beverages. A higher shared meal frequency was also related to a higher intake of many (12/21) nutrients (e.g., protein, dietary fiber, and potassium). However, in women, there was a positive association between shared meal frequency and sodium intake. A higher frequency of household shared meals may be related to a more favorable dietary intake; except for concerns about sodium intake in women.

## 1. Introduction

Several review articles have reported the potential benefits of having more frequent family meals in the past decade. These benefits have been reported in psychosocial areas (e.g., eating disorder) [1,2,3] and family functioning (e.g., family communication) [4] as well as dietary intake (e.g., a higher intake of fruits and vegetables and lower intake of sugar-sweetened beverages) [2,4,5,6]. However, the studies included in these reviews were primarily conducted in Western countries, with participants limited to children, adolescents, or households with children and adolescents.

Among US adults, only a few studies have examined the relationship between the frequency of household shared meals and a limited number of dietary variables [7,8,9,10,11]. These studies showed that a higher frequency of meals shared with household members was positively related to the intake of fruits and vegetables [8,9,10,11]; however, the results for sugar-sweetened beverage intake were not consistent [7,9,11]. Furthermore, a study conducted in adults aged 20–31 years suggested that the association between dietary intake and frequency of shared meals may differ among men and women [7]. All studies were conducted in parents (mean age, 31–42 years) with children aged <18 years [8,9,10] or young adults (mean age, 19–25 years) [7,11]. These studies did not include older age groups (e.g., ≥60 years) or those from households without children; thus, results obtained from these studies had limited representativeness.

In Japan, eating together (*Kyōshoku*) with family members is highly valued as a mean to promote physical and social wellbeing [12]. The promotion of eating together was included in the Fourth Basic Plan for the Promotion of *Shokuiku* (Food and Nutrition Education), developed under the Basic Act on *Shokuiku* set by the Japanese government in 2005 [13]. Although a few Japanese studies have examined the relationship between family meals and dietary outcomes [14], few have targeted adult populations, and none have quantified dietary intake [15,16].

To the best of our knowledge, all studies that examined the relationship between the frequency of shared meals with family members and dietary intake were based on self-report questionnaires. Shared meal frequency was usually measured as ‘family meals’, which depended on participant perceptions. Food groups and nutrients assessed in previous studies were limited in number and may not reflect the total diet [7,8,9,10,11,15,16]. In the National Health and Nutrition Survey in Japan (NHNSJ), which covered the general population with various characteristics, dietary intake is assessed based on the combination of food weighing and the approximated shared proportion method (for shared foods) using a one-day dietary record at the household level. Data from these dietary records allow researchers to define shared meals based on the actual intake information (i.e., foods applied by approximated shared proportion) and to assess total one-day dietary intake from a broader range of food groups and nutrients. Therefore, this study aimed to explore the relationship between daily household shared meal frequency and the intake of food groups and nutrients in adults aged ≥20 years living with household members recruited in the 2018 and 2019 NHNSJ.

## 2. Methods

### 2.1. Data Source and Analytic Sample

This study was based on secondary analyses of the 2018 and 2019 NHNSJ [17,18]. The NHNSJ is a cross-sectional household interview and examination survey conducted annually in November, which intends to represent free-living non-institutionalized populations aged ≥1 year across 47 prefectures in Japan. The NHNSJ includes three sub-surveys: dietary survey, physical examination, and lifestyle questionnaires. The sampling of the NHNSJ follows a stratified two-stage cluster design. In the first stage, a simple random sample of the census enumeration areas is drawn from each prefecture. In the second stage, census enumeration areas are divided into units containing 20–30 households. By randomly selecting units from each prefecture, all households and household members from 300 unit blocks (296 for the 2019 survey because of the typhoon strike) are included as potential samples for the NHNSJ [17,18,19].

**Figure 1:**
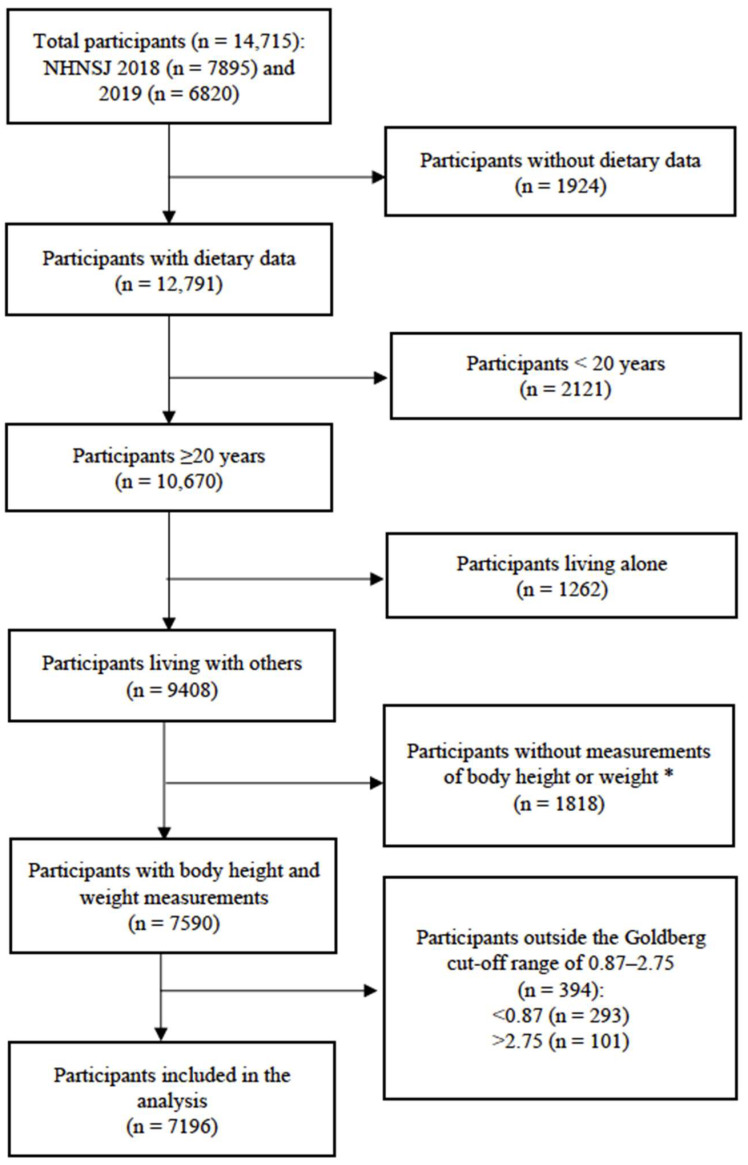
Flowchart of the analytical sample of this study. * Excluded for calculating BMR to assess misreporting of energy intake.

Adults aged ≥20 years were targeted in this study. Individuals living alone were considered to have a low probability of having household shared meals; therefore, they were excluded from the current analysis (Figure 1). Participants without body height or weight measurements were excluded (n 1818). Energy intake (EI) misreporting may be related to both meal frequency (and shared meal frequency) and dietary intake. Thus, the Goldberg cut-off (based on the 95% confidence interval of the EI to basal metabolic rate (BMR) ratio) was used to exclude under- or over-reporters [20]. BMR was calculated based on a formula developed and validated for the Japanese population [21,22]. The cut-off range applied in this study was 0.87–2.75, which was set for sedentary (i.e., physical activity level of 1.55) individuals with a one-day dietary intake available [20]. In total, 7196 participants were included in the analysis.

### 2.2. Dietary Assessment

Dietary data were collected through a one-day, semi-weighed dietary record as part of a nutrition survey conducted by the NHNSJ. Household meal preparers, typically women, recorded the dietary intake of each household member on a selected day. The recording day avoided Sundays, public holidays, or atypical eating occasions (e.g., weddings) to reflect regular eating habits. Additionally, they gathered information from other household members for meals not eaten together, ensuring a comprehensive record of individual food consumption.

**Figure 2:**
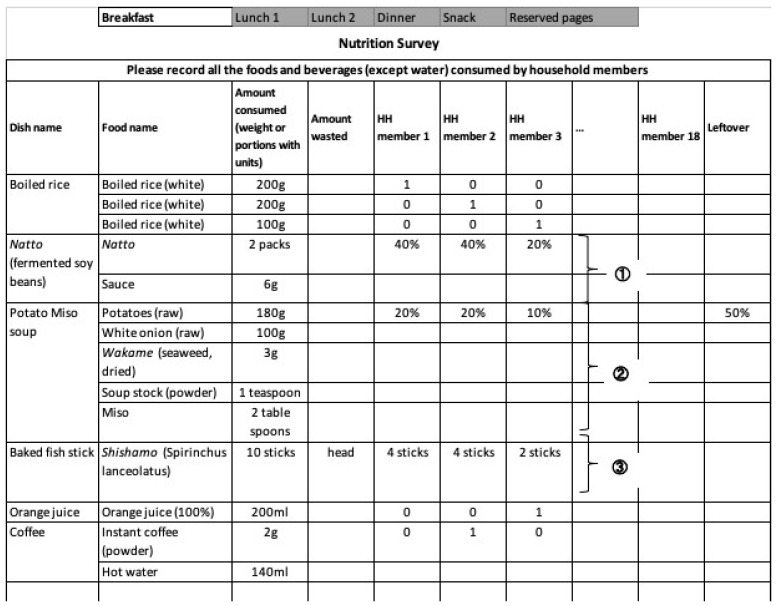
An example of the 1-day dietary record of breakfast to be filled by the meal preparers used in the National Health and Nutrition Survey in Japan. Intakes of the dishes ①②③ were assumed based on the approximated shared proportion method, which would later calculate the intake of the component ingredients. For example, the intake value of the food item Natto for HH member 1 would be calculated as 80 g (based on 40 g/pack) × 40% = 32 g. HH, household. The amount consumed means the total amount of food, not including waste or inedible parts (e.g., fish head, peels).

Trained interviewers (mainly registered dietitians) visited each sample household to distribute the dietary record booklet and guide meal preparers with written and verbal instructions. The booklet consisted of four sections: breakfast, lunch, dinner, and snacks. Meal preparers were instructed to weigh food items whenever feasible or estimate portion sizes using household measures such as spoons, cups, and bowls. An approximated shared proportion for shared food was allocated to each member’s meal intake, which involved shared dishes with mixed ingredients. Nevertheless, records were encouraged to be kept separate for each person for foods consumed individually (e.g., boiled rice) (Figure 2). Data on food waste and leftovers during preparation and consumption were recorded.

To enhance the quality and reliability of dietary records, detailed information on meals consumed outside the home was requested. The information included the portion size consumed, the restaurant’s or manufacturer’s name where the meal was prepared, and details on the ingredients and quantities used or consumed, for instance, through menus, recipes, and packaging. When a household member failed to provide information on their dietary intake for a specific meal, they were excluded from the analysis.

After completing the dietary records, a two-stage confirmation process was implemented to ensure accuracy and completeness. Initially, trained interviewers revisited each household, typically on the following weekday, to collect the dietary records. During the visit, they scrutinized the record for the accuracy of the recorded approximated shared proportion, looked for any missing or incorrect entries, and asked for additional details on recorded dietary information, such as specifics about meals eaten outside the home. The interviewers then carefully processed the data by coding food items according to the Standard Tables of Food Composition in Japan (STFCJ, 2015) [23], applying cooking codes (i.e., baking and boiling) (for calculating nutrient intake as consumed), and assigning weights of food items recorded in household measures or portion sizes. The weight assignment was guided by Japanese cookery manuals, which had thorough information on several aspects of cooking practice, including but not limited to converting common household measurements to weights, adjusting oil absorption, and converting dry weight to wet weight.

In the second stage, research dietitians at the National Institute of Health and Nutrition conducted a detailed review of the dietary records. This involved verifying the accuracy and appropriateness of the food codes applied to each item and scrutinizing any unusual intake amounts or overlooked ingredients. Discrepancies or concerns were resolved through consultation with the interviewers.

In this study, food items were classified into 17 major groups according to the NHNSJ categorization, which includes cereals, potatoes, sugars, pulses, nuts, vegetables, fruits, mushrooms, seaweeds, fish, meat, eggs, dairy, fats and oils, confectionaries, beverages, and condiments, based on the STFCJ [23]. Energy and 21 nutrients were included in the analysis. Nutrient intake from dietary supplements was excluded due to the absence of a standardized food composition table for these items.

The relative accuracy of one-day household-based dietary records has been previously assessed [24]. Female dietetic students (n 32) and their mothers (n 32) participated in the study. The reference method was individual-based dietary records taken by students specifically trained to take the records. The mothers, usually meal preparers, were asked to take the household-based dietary record, which was used for comparison with the individual-based dietary records. The household-based method underestimated energy, protein, fat, and carbohydrate by 6.2%, 5.7%, 6.7%, and 6.3%, respectively, compared to the individual-based method [24].

### 2.3. Definition of Household Shared Meal Occasions

In this study, we used the term ‘shared meal’ instead of ‘family meal’ due to the lack of specific contextual factors (e.g., eating location, presence of persons, and meal duration) [25] in NHNSJ. A ‘shared meal’ refers to any main meal (breakfast, lunch, or dinner) where food items are divided among household members according to the approximated shared proportion (meaning a food item was divided by at least two household members) recorded in the dietary record. We focused on thirteen food groups to identify a shared meal occasion: cereals, potatoes, pulses, nuts, vegetables, fruits, mushrooms, seaweeds, fish, meat, eggs, dairy, and confectioneries. We excluded sugars, fats and oils, beverages, and condiments from this classification because they are not typically main meal components. A meal was classified as a shared meal occasion if at least one item from the specified food groups was consumed in a shared proportion (assigned with approximated shared proportion described above), irrespective of the amount or whether the food item was consumed more than once. Based on the frequency of shared meals, participants were categorized into four groups: 0, 1, 2, and 3 times per day.

### 2.4. Covariates

Based on previous studies, age, occupation, household size, and snacking were included as covariates [26,27,28,29]. Meal skipping was also included because it may be related to both meal frequency (and possibly shared meal frequency) and dietary intake [30]. Residential area characteristics were included because, in a previous Japanese study, it was reported to have a significant relationship with vegetable intake (and thus maybe intake of other food groups as well) [31]. Information on sex (male or female), age, and occupation was obtained from self-report questionnaires. Age was categorized into seven groups: 20–29, 30–39, 40–49, 50–59, 60–69, 70–79, and ≥80 years. Occupation was initially queried using a 12-group classification but further combined into four groups in this study based on distributions according to shared meal frequency and the similarities between occupations. The four groups of occupations were ‘office/service’ (including professional, managerial, clerical, sales, and service workers), ‘manual’ (including security workers, transportation and machinery operators, and production process workers), ‘agriculture-related’ (including agricultural, forestry, and fishery workers), and ‘housework/other’ (including housework, other, and students). Household size was assessed by eligible household members who participated in at least one of the three sub-surveys of the NHNSJ. Snacking (i.e., yes or no) was determined based on whether any food item (without exclusion of food groups) was recorded in the snack section. Meal skipping was determined based on self-reported meal types for each main meal occasion (breakfast, lunch, and dinner); this included ‘no’ if one had all main occasions described as ‘home-cooked’, ‘take-out’, ‘dining out’, ‘provided-meal by schools or nursery facilities’, ‘provided-meal by work facility’, or ‘sweets/fruit/dairy/beverages only’, and ‘yes’ if ≥1 occasion was described as ‘dietary supplements only’ or ‘nothing’. Residence area characteristics were identified following the classification of the NHNSJ (i.e., government-designated cities, cities with populations of >150,000, 50,000–150,000, <50,000, or town/village) [17,18].

### 2.5. Statistical Analysis

This study analyzed men and women separately because between-sex differences were observed for most intakes of food groups and nutrients, which was also suggested in a previous study [7]. All analyses were performed using SAS version 9.4 (SAS Institute). Statistical significance was defined as a two-tailed *p*-value of <0.05. Basic characteristics were expressed as means and standard deviations (SD) for continuous variables and numbers and percentages for categorical variables across the shared meal frequencies. The analysis of variance (ANOVA) was used to test the statistical differences across groups, and the Mantel–Haenszel chi-square test was used for categorical variables.

The intakes of the 21 nutrients and 17 food groups (including sugars, fats and oils, beverages, and condiments) were analyzed based on the density method (as % EI for protein, total fat, SFA, and carbohydrate, and unit per 1000 kcal for food groups and other nutrients) [32]. As dietary intake information may be obtained from ≥1 household member of the same sex group, multilevel linear regressions were used to adjust the mean intake of food groups and nutrients by including households as a random effect. Fixed effects were adjusted for all covariates described in the previous section. Only consumers (with intake >0 g/day) were included in analyzing the food groups’ intake. The percentage of consumers in each food group was calculated and compared across the shared meal frequency based on Wald chi-square tests from multivariate logistic regression. Nutrient intake was analyzed for all participants because nearly all participants consumed all of the selected nutrients. Between-group differences in dietary intake across shared meal frequencies were compared by ANOVA and trend tests based on linear contrasts to examine the linear relationship between shared meal frequency and dietary intake.

### 2.6. Ethics Statement

No institutional review board approval was required because this study was based on de-identified secondary data analyses according to the Ethical Guidelines of Epidemiological Research by the Ministry of Education, Culture, Sports, Science and Technology and the Ministry of Health, Labour and Welfare.

## 3. Results

The study included 3310 men and 3886 women. The mean age was 58.1 (SD, 17.1) years for men and 56.9 (SD, 16.7) years for women. The mean number of food items consumed in a day from the designated 13 food groups (excluding sugars, fats and oils, beverages, and condiments) was 28.7 (SD, 10.0) and 27.9 (SD, 10.0) in men and women, respectively, of which 64% (men) and 67% (women) of the food items were consumed as shared food items. Participants’ characteristics are shown in Table 1. About 80% of men and women had two or more shared meals on the recording day. The number of food items consumed and the proportion of shared items increased across shared meal frequencies. Participants aged ≥60 years were more likely to have three shared meals (58.4%) than the younger age group (20–49 years) (23.3%). Participants with occupations under ‘office/service’ and ‘manual’ were less likely to have three shared meals (28.1%) than those who had agricultural-related occupations or mainly doing housework (66.3%). Men without snacking had a significantly higher frequency of shared meals. Compared with the participants included in the present analysis, those excluded from this analysis had low mean values of the number of consumed food items, number of shared meal occasions, and total EI (Supplemental Table S1). Additionally, participants excluded from the present analysis were more likely to be men and younger in age (<50 years), had occupations under ‘office/service’ and ‘manual’, and lived with more household members. They were also more likely to be meal skippers and to have no snacks.

Table 2 shows the multivariate-adjusted mean intake of energy (kcal) and nutrients (% total energy or unit/1000 kcal) by the category of shared meal frequency. Shared meal frequency was positively related to EI in women but not in men. The shared meal frequency showed a positive linear relationship with the intake of most nutrients, namely, protein, total fat (only men), dietary fiber, potassium, calcium (only men), magnesium, iron, zinc, copper, niacin, folate, vitamins K, B1, B6, B12 (only women), and C (only men), and sodium (only women). Moreover, having more frequent shared meals was related to a lower SFA intake in women. 

For the percentage of consumers, both men and women showed higher values for pulses, nuts, fruit, mushrooms, fish, meat, eggs, and fats and oils with an increased shared meal frequency. No significant differences were observed for sugars, confectionaries, and beverages (Table 3). Table 4 shows the multivariate-adjusted mean intakes (g/1000 kcal) of food groups in consumers with intake >0 g according to household shared meal frequency. Almost all participants consumed cereals, vegetables, and condiments on the recording day. Overall, the frequency of shared meals revealed positive linear relationships with the intakes of potatoes, vegetables, mushrooms, and condiments and inverse relationships with confectioneries and beverages. In men, the shared meal frequency was positively related to the intake of pulses and eggs, while it was inversely related to cereals and fats and oils. In women, the frequency of shared meals was positively related to seaweed intake, while it was inversely related to the intake of sugars, fruits, and dairy.

## 4. Discussion

To the best of our knowledge, this is the first study to examine the relationship between household shared meal frequency and dietary intake based on dietary records obtained from national surveys. This study revealed that individuals with more household shared meals per day had a higher intake of potatoes, vegetables, mushrooms, and condiments with a more favorable nutrient (e.g., protein, dietary fiber, and most micronutrients) intake profile. However, in women, there was a positive association between shared meal frequency and sodium intake.

The majority of participants (men, 79%; women, 83%) living with ≥1 household members included in this study had at least one shared meal on the recording day, which was substantially higher than the corresponding values observed in Nordic countries (54–64%) [33] and the US (49–64%) [25]. The discrepancies in shared meal frequency from the Western countries were somewhat in line with the finding from a US study, which reported that Asian Americans had a higher frequency of family meals (5.3 family meals/week) than other ethnic/racial groups (4.1–4.5 family meals/week) [27]. In Japan, according to the results obtained from the Attitude Survey on Food and Nutrition Education (hereinafter referred to as the Attitude Survey) conducted by the Japanese government, the habitual frequencies of having family breakfast and dinner were 57% and 67% for adults aged ≥20 years and living with other household members, respectively [34,35]. Thus, the result could be interpreted as a frequency of having family breakfast and dinner about 1.2 times/day (i.e., the summation of 0.57 and 0.67), which is lower than the mean frequency of 1.7 times (the sum of shared breakfast and dinner) calculated in our study. The Attitude Surveys also reported that 5.6% of the adult participants did not share meals with their family members [34,35], slightly higher than the value observed in this study (3.1%). In addition to differences in methods (i.e., self-report questionnaire vs. dietary record) used to assess shared meal frequency, the discrepancies from previous reports may also be explained by the fact that the shared meal frequency calculated in our study was based on a one-day dietary record, which cannot reflect one’s habitual intake. Another reason could be the different definitions used for family/shared meals. Contrary to the definitions of family meals used in the previous analyses mentioned above [25,27,33,34,35], which included contextual information (e.g., family members’ presence at a meal), we defined household shared meals solely based on dietary content shared with other household members. In the case of this study, shared meal frequency may be overestimated when meal contents are the same for multiple household members but may be consumed at different locations (e.g., packed lunch box) or times (e.g., separately plated dinner).

Previous studies based on single questions [8,10] or FFQs [7,9,11] showed a consistent positive relationship between family/shared meal frequency and fruit and vegetable intake. Despite the differences in methods used for assessing dietary intake (one-day dietary record vs. questionnaire) and basic characteristics of participants (20–79 years vs. young adults or parents), our study was consistent with previous studies showing that shared meal frequency is positively related to vegetable intake [8,9,10,11]. Although findings on other food groups were limited in previous studies, the higher vegetable intake to shared meal frequency suggests that shared meals may contain more ingredients required for cooking than meals eaten alone [36]. This interpretation may also be reflected in our study that an increased shared meal frequency was also related to a higher intake of condiments; a higher percentage of consumers of fish, meat, eggs, fats, and oils; and a higher intake and percentage of consumers for potatoes, pulses, and mushrooms. The foods listed above require some form of cooking for consumption.

At the nutrient level, a higher protein, dietary fiber, and most micronutrients were related to an increased frequency of shared meals, which may imply that food choices made for sharing with household members may be more health-conscious than those made when eating alone. In addition, family meal frequency is positively related to the proportion of home-cooked meals [37], although not directly comparable. A UK study showed that the proportion of EI from home-prepared food was positively related to diet quality [38]. Moreover, a Japanese study showed that an increased frequency of eating out was related to higher odds of having an inadequate intake of dietary fiber, vitamin C, and several minerals [39]. However, our study showed that an increased shared meal frequency was related to a higher sodium intake in women, which was somewhat in line with a previous study that showed that home-prepared foods were the major source of sodium intake in Japan [40].

In this study, we observed several differences between the sexes. In participants with a shared meal at zero or once per day, >60% of women consumed fruits with a mean intake of 83–98 g/1000 kcal/day, compared with approximately 40% in men with a mean intake of 56–68 g/1000 kcal/day. In addition, although the amount of dairy intake did not differ between shared meal frequencies in men, an increased percentage of consumers was observed across the frequency groups (60% [once/day] − 77% [three times/day], *p* = 0.0002). In women, however, although the percentage of consumers remained unchanged (73% [one time/day] − 80% [three times/day], *p* = 0.37) across the frequency groups, participants in the zero time/day group had a significantly higher intake than the other three groups (based on a *post hoc* Tukey test). The differences in food group intake were consistent with those observed at the nutrient level. For example, a negative relationship between shared meal frequency and SFA in women may reflect a lower intake of dairy and confectioneries in the higher frequency group, and a higher vitamin C intake according to shared meal frequencies in men was consistent with a higher fruit intake across shared meal frequencies. These findings suggest a different eating pattern between men (e.g., a higher intake of cereals, fats and oils, and confectionaries) and women (e.g., higher fruit, dairy, sugars, and confectionaries) without shared meals.

Furthermore, differences in sodium intake to the shared meal frequency were observed between men and women. In men, although condiments (the largest food source for sodium intake) [41] increased with a shared meal frequency, sodium intake did not. One interpretation would be that the same condiment was consumed across the shared meal frequencies, and sodium intake from other food sources decreased in men; this is possible given that the intake of cereals (including bread and instant noodles) and confectioneries (including savory snacks) was reduced across the shared meal frequencies. Another interpretation is that condiment intake may be underestimated in the lower frequency groups due to proxy-reporting bias, given that the household meal preparers (usually women) [42] took the dietary records. Also, when one had a meal not prepared by the meal preparer, food intake must be reported from one’s recall, which may not accurately assess intakes from condiments [43]; in this case, standardized recipe codes may be applied.

We also noted that participants in their 20s–50s and with occupations characterized as ‘office/service’ or ‘manual’ were more likely to have household shared meals two times/day rather than three times, as most were likely to work outside the home. Work style is one of the most critical factors for household shared lunch meals [29]. Although an increased frequency of household-shared meals may be related to a more favorable dietary intake regardless of age or occupation, our study showed that promoting healthy eating by encouraging more household-shared meals may not be practical in some populations [44].

### Limitations

This study has several limitations that need to be noted. First, the definition of household shared meal occasion used in this study did not consider food weight consumed but was based on whether more than or equal to one food item from the designated 13 food groups was shared with other household members. However, no evidence exists to determine a cut-off value for classifying shared meal occasions based on food weight. Moreover, we excluded sugars, fats, oils, condiments, and beverages, which are seldom used as the main ingredients for a meal. Future studies may need to incorporate food weights into the definition and generate a different definition for each meal occasion due to different dietary contents and the amount consumed [45,46].

Second, although the NHNSJ intended to represent the non-institutionalized Japanese population aged ≥1 year, only 64.9% (2018) [17] and 63.5% (2019) [18] of the sampled households participated. Unfortunately, no information was obtained from non-participating households. Participants excluded from this study were more likely to be men, younger, and meal skippers. These factors were negatively related to the shared meal frequency observed in our study and a previous Japanese study [29] and are also known to be negatively related to dietary quality observed in other Japanese studies [30,47]. Thus, the relationship between the shared meal frequency and dietary intake observed in this study may be conservative.

Third, the one-day dietary records may not accurately represent the habitual frequency of having shared meals. In addition, as most participants were classified as having more than or equal to two shared meals a day, fewer observations in the lower frequency group (i.e., zero and one time a day) may reduce power for detecting the differences in dietary intake across the groups.

Fourth, measurement errors in dietary intake could not be avoided. Although we used energy-adjusted intake for nutrients and foods [32] and excluded misreporting by using the Goldberg cut-off, bias from misreporting may still remain due to a lack of reliable biomarkers that would be feasible to apply in this study [20].

Fifth, previous studies have shown that socioeconomic factors are related to family/shared meal frequency and diet quality [26,48]. Two important covariates (i.e., education and household income) could not be adjusted for in this analysis due to a lack of information: data on education was unavailable for all NHNSJ, and data on household income was unavailable for the NHNSJ 2019. Future NHNSJ should consider including these variables.

Sixth, this was a cross-sectional analysis, from which the causal relationship between shared meal frequency and dietary intake could not be inferred. Despite no currently available longitudinal studies conducted in adults, longitudinal studies conducted in children or adolescents showed positive and negative relationships with fruit intake and snacks, respectively [4].

## 5. Conclusions

This study identified household shared meals based on the actual dietary intake obtained from a one-day dietary record in national nutrition surveys in Japan. As a result, Japanese adults with a higher frequency of household shared meals showed a more favorable intake of several food groups and most nutrients. However, they were also related to a higher sodium intake in women.

## Figures and Tables

**Table 1:** Participants’ basic characteristics according to the household shared meal frequency in Japanese adults ≥20 years living with ≥1 household member from the 2018 and 2019 NHNSJs (n = 7196) *.

		Men						Women					
			0 Times/Day (n 115)	1 Time/Day (n 586)	2 Times/Day (n 1215)	3 Times/Day (n 1394)	*p* †		0 Times/Day (n 109)	1 Time/Day (n 533)	2 Times/Day (n 1481)	3 Times/Day (n 1763)	*p* †
			Mean (SD)						Mean (SD)				
Number of consumed food items ‡			24.2 (10.1)	24.6 (9.1)	28.2 (9.3)	31.2 (10.2)	<0.0001		23.6 (9.6)	24.1 (8.89)	27.1 (9.3)	30.1 (10.3)	<0.0001
Proportion of shared food items §			0% (0%)	41% (19%)	60% (18%)	83% (15%)	<0.0001		0% (0%)	42% (19%)	61% (18%)	84% (14%)	<0.0001
BMI (kg/m^2^)			24.4 (3.50)	23.7 (3.44)	23.8 (3.40)	23.7 (3.21)	0.21		22.0 (3.65)	22.3 (3.83)	22.3 (3.61)	22.6 (3.51)	0.042
		N	%					N	%				
		3310	3.5	17.7	36.7	42.1	<0.0001	3886	2.8	13.7	38.1	45.4	<0.0001
Age group (years)							<0.0001						<0.0001
	20–29	216	3.2	23.1	43.1	30.6		240	1.7	19.2	46.7	32.5	
	30–39	337	5.0	29.7	44.8	20.5		450	2.4	17.8	46.4	33.3	
	40–49	533	3.9	28.1	45.8	22.1		651	1.8	18.1	49.0	31.0	
	50–59	472	4.0	22.2	48.5	25.2		692	4.6	17.2	42.3	35.8	
	60–69	738	3.0	16.0	33.9	47.2		847	2.2	11.2	31.8	54.8	
	70–79	730	2.7	7.1	25.8	64.4		714	2.9	6.2	26.5	64.4	
	≥80	284	3.2	3.9	21.1	71.8		292	3.4	10.6	30.8	55.1	
Occupation ||							<0.0001						<0.0001
	Office/service	1396	3.7	22.2	45.1	29.0		1684	3.5	17.3	46.2	33.0	
	Manual	697	4.6	28.7	40.5	26.3		210	1.9	15.2	47.1	35.7	
	Agriculture-related	227	3.5	5.3	22.9	68.3		139	1.4	2.9	22.3	73.4	
	Housework/other	990	2.3	6.5	25.5	65.8		1853	2.4	11.1	30.9	55.6	
Household size (persons) ¶							0.0007						0.006
	2	1377	3.8	14.7	31.2	50.3		1573	4.5	15.5	33.1	47.0	
	3	893	4.6	18.8	40.6	35.9		1060	2.1	13.2	40.7	44.1	
	4	607	2.8	23.9	41.8	31.5		732	1.5	13.1	42.6	42.8	
	≥5	433	1.2	16.2	39.0	43.6		521	1.2	10.2	41.8	46.8	
Meal skipping **							<0.0001						<0.0001
	Yes	184	6.5	51.1	42.4	-		157	8.3	43.9	47.8	-	
	No	3126	3.3	15.7	36.4	44.6		3729	2.6	12.4	37.7	47.3	
Snacking ††							<0.0001						0.27
	Yes	1674	3.8	20.1	42.4	33.8		1453	2.9	14.7	37.8	44.7	
	No	1636	3.2	15.3	30.9	50.7		2433	2.8	13.2	38.3	45.8	
Characteristics of residential areas							<0.0001						<0.0001
	Government-designated cities	579	4.0	18.0	37.5	40.6		716	3.8	14.7	39.4	42.2	
	Population ≥150,000	1072	5.2	19.6	36.3	38.9		1261	2.9	14.0	39.7	43.3	
	Population 50,000–150,000	1043	2.3	18.5	37.5	41.7		1200	2.4	13.9	38.7	45.0	
	Population <50,000	208	2.4	14.9	32.7	50.0		252	2.4	14.3	29.8	53.6	
	Town/village	408	1.7	11.8	36.8	49.8		457	2.2	10.5	34.8	52.5	

BMI, body mass index; NHNSJ, National Health and Nutrition Survey in Japan; SD, standard deviation. * Meals included breakfast, lunch, and dinner, but not snacks. Shared meal refers to a meal occasion (i.e., breakfast, lunch, and dinner) with ≥1 food item (not included foods included in ‘sugars’, ‘fats and oils’, ‘beverages’, and ‘condiments’) that was consumed by ≥1 household member based on the application of the approximated shared proportion. † ANOVA for continuous variables; Mantel–Haenszel χ^2^ test for categorical variables. ‡ Food consumed at breakfast, lunch, and dinner was included in the calculation. Food items are the basic food or ingredient units used to calculate nutrient intake. Food items were excluded if they belonged to the following food groups ‘sugars’, ‘fats and oils’, ‘beverages’, or ‘condiments’. Duplication and the amount of intake were not considered when calculating the number of consumed food items. For example, if one consumed an apple each at breakfast and dinner or cabbage from salad dishes and sauteed vegetables at dinner, then the food item apple or cabbage would be counted twice. § Calculated as shared food items from food groups excluding ‘sugars’, ‘fats and oils’, ‘beverages’, or ‘condiments’ divided by the number of consumed food items calculated above regardless of the amount of intake or duplication. || ‘Office/service’ included professional, managerial, clerical, sales, and service workers; ‘manual’ included security workers, transportation and machinery operators, and production process workers; ‘agriculture-related’ included agricultural, forestry, and fishery workers; ‘housework/other’ included housework, other, and students. ¶ Based on household members aged ≥1 year who participated in a ≥1 sub-survey consisting of the NHNSJ (i.e., the nutrition survey, physical examination survey, and lifestyle questionnaire survey). ** ‘Yes’ is defined based on meal types that were classified by participants as ‘dietary supplements only’ or ‘no consumption’ for breakfast, lunch, and dinner. ‘No’ is defined based on meal types that were classified by participants as ‘home-cooked’, ‘take-out’, ‘dining out’, ‘provided-meal by school or nursery facility’, ‘provided-meal by work facility’, or ‘sweets/fruits/dairy/beverages only’. †† Determined by whether any food or beverage was recorded under the snack section of the dietary record booklet.

**Table 2:** Intake of energy and nutrients by household shared meal frequency in Japanese adults ≥20 years living with ≥1 household member from the 2018 and 2019 NHNSJs (n = 7196) *.

		Men										Women									
		0 Times/Day (n 115)		1 Time/Day (n 586)		2 Times/Day (n 1215)		3 Times/Day (n 1394)				0 Times/Day (n 109)		1 Time/Day (n 533)		2 Times/Day (n 1481)		3 Times/Day (n 1763)			
	Unit	Mean	SE	Mean	SE	Mean	SE	Mean	SE	*p* for ANOVA	*p* for Trend †	Mean	SE	Mean	SE	Mean	SE	Mean	SE	*p* for ANOVA	*p* for Trend †
Energy	kcal	2072	49	2080	27	2101	24	2158	25	0.007	0.06	1519	40	1512	23	1599	20	1642	21	<0.0001	<0.0001
Protein	%E	13.1	0.29	14.0	0.15	14.5	0.14	14.8	0.14	<0.0001	<0.0001	14.3	0.3	14.9	0.17	15.3	0.15	15.6	0.16	<0.0001	<0.0001
Total fat	%E	26.2	0.72	25.9	0.39	26.6	0.35	27.5	0.36	0.0003	0.03	29.1	0.8	28.6	0.44	28.8	0.38	28.7	0.39	0.90	0.58
SFA	%E	7.7	0.27	7.6	0.15	7.6	0.13	7.8	0.14	0.56	0.95	8.9	0.3	8.4	0.17	8.3	0.15	8.0	0.16	0.002	0.002
Carbohydrate	%E	53.4	0.92	52.9	0.50	52.9	0.45	52.8	0.47	0.91	0.47	53.2	0.9	53.4	0.51	52.9	0.45	53.0	0.46	0.67	0.64
Dietary fiber	g/1000 kcal	6.4	0.27	7.3	0.15	7.8	0.13	8.3	0.14	<0.0001	<0.0001	7.9	0.3	8.9	0.18	9.0	0.16	9.5	0.16	<0.0001	<0.0001
Sodium	g/1000 kcal ‡	4.9	0.16	5.0	0.09	5.1	0.08	5.0	0.08	0.10	0.25	5.1	0.18	5.4	0.10	5.4	0.09	5.5	0.09	0.01	0.01
Potassium	mg/1000 kcal	931	32	1015	17	1069	16	1142	16	<0.0001	<0.0001	1162	38	1205	22	1234	19	1300	20	<0.0001	<0.0001
Calcium	mg/1000 kcal	199.0	9.9	201.3	5.4	217.9	4.9	231.3	5.0	<0.0001	0.0002	251.2	12.3	248.3	7.1	257.9	6.2	266.1	6.4	0.02	0.13
Magnesium	mg/1000 kcal	105.9	4.0	119.0	2.1	124.8	1.9	129.7	2.0	<0.0001	<0.0001	125.3	4.4	132.3	2.5	138.6	2.2	143.1	2.3	<0.0001	<0.0001
Iron	mg/1000 kcal	3.2	0.11	3.5	0.06	3.8	0.1	4.0	0.06	<0.0001	<0.0001	3.8	0.1	4.1	0.08	4.3	0.1	4.5	0.07	<0.0001	<0.0001
Zinc	mg/1000 kcal	3.9	0.11	4.1	0.06	4.3	0.05	4.3	0.05	<0.0001	<0.0001	4.2	0.1	4.3	0.06	4.5	0.05	4.6	0.06	<0.0001	0.0004
Copper	mg/1000 kcal	0.5	0.014	0.5	0.008	0.6	0.007	0.6	0.007	<0.0001	<0.0001	0.57	0.0	0.60	0.009	0.62	0.008	0.63	0.008	<0.0001	<0.0001
Vitamin A	μgRAE/1000 kcal	292.1	48.2	255.7	26.1	278.8	23.5	286.8	24.2	0.66	0.96	300.7	41.1	325.9	23.1	334.8	20.3	352.1	20.9	0.34	0.17
Vitamin K	μg/1000 kcal	81.2	8.7	94.9	4.7	113.2	4.3	129.3	4.4	<0.0001	<0.0001	122.5	10.8	125.8	6.2	141.1	5.5	154.4	5.6	<0.0001	0.0004
Vitamin B1	mg/1000 kcal	0.41	0.02	0.46	0.01	0.46	0.01	0.48	0.01	<0.0001	<0.0001	0.47	0.02	0.49	0.01	0.51	0.01	0.51	0.01	0.01	0.003
Vitamin B2	mg/1000 kcal	0.55	0.02	0.53	0.01	0.55	0.01	0.57	0.01	0.001	0.13	0.65	0.02	0.62	0.01	0.63	0.01	0.64	0.01	0.03	0.81
Niacin	mg/1000 kcal	14.0	0.41	15.4	0.22	15.5	0.20	15.9	0.21	<0.0001	<0.0001	15.0	0.4	15.9	0.25	16.2	0.22	16.7	0.22	<0.0001	<0.0001
Vitamin B6	mg/1000 kcal	0.49	0.02	0.56	0.01	0.57	0.01	0.61	0.01	<0.0001	<0.0001	0.57	0.02	0.60	0.01	0.62	0.01	0.65	0.01	<0.0001	<0.0001
Vitamin B12	μg/1000 kcal	2.9	0.34	2.9	0.18	3.4	0.16	3.2	0.17	0.04	0.12	2.8	0.4	3.0	0.20	3.4	0.17	3.6	0.18	0.001	0.005
Folate	μg/1000 kcal	117.7	6.5	122.5	3.5	136.6	3.2	146.2	3.3	<0.0001	<0.0001	155.1	7.2	159.6	4.1	166.0	3.6	175.2	3.7	<0.0001	0.001
Vitamin C	mg/1000 kcal	30.1	2.9	35.1	1.6	40.2	1.4	46.2	1.5	<0.0001	<0.0001	58.1	3.8	47.5	2.2	49.8	1.9	54.9	2.0	<0.0001	0.50

NHNSJ, National Health and Nutrition Survey in Japan; SE, standard error; SFA, saturated fatty acids; RAE, retinol activity equivalents.* Shared meal refers to a meal occasion (i.e., breakfast, lunch, and dinner) with ≥1 food item (not included food groups of ‘sugars’, ‘fats and oils’, ‘beverages’, and ‘condiments’) that was consumed by ≥1 household member based on the application of the approximated shared proportion. Mean intake values were adjusted based on multilevel linear regression, including age groups (i.e., 20–29, 30–39, 40–49, 50–59, 60–69, 70–79, and ≥80 years), occupation (i.e., office/service, manual, agriculture-related, and housework/other), meal skipping (yes or no), snacking (yes or no), and characteristics of residence area (i.e., government-designated cities, population ≥150,000, 50,000–150,000, and <50,000, and town/village) as fixed effects and households as a random effect. † Based on linear contrasts across frequencies of shared meal occasions. ‡ Salt equivalent (g) = Na (mg) × 2.54/1000.

**Table 3:** Percentages of consumers by household shared meal occasions in Japanese adults ≥20 years living with ≥1 household member from 2018 and 2019 NHNSJ (n 7196) *.

	Men					Women				
	0 Time/Day	1 Time/Day	2 Times/Day	3 Times/Day		0 Time/Day	1 Time/Day	2 Times/Day	3 Times/Day	
Food Groups	n 115	n 585	n 1215	n 1394	*p* †	n 109	n 533	n 1481	n 1763	*p* †
Cereals	100.0%	99.8%	100.0%	100.0%	na	99.1%	99.4%	99.7%	99.9%	na
Potatoes	68.7%	71.8%	71.3%	74.0%	0.27	62.4%	65.9%	69.6%	73.1%	0.02
Sugars	72.2%	76.8%	80.7%	79.3%	0.055	73.4%	74.7%	77.2%	78.7%	0.52
Pulses	54.8%	62.1%	76.9%	83.8%	<0.0001	62.4%	69.0%	78.3%	81.3%	<0.0001
Nuts	12.2%	24.1%	28.9%	34.8%	<0.0001	33.0%	25.1%	31.1%	33.5%	0.004
Vegetables	99.1%	100.0%	99.9%	100.0%	na	96.3%	99.6%	99.9%	99.9%	na
Fruits	39.1%	40.8%	55.2%	74.6%	<0.0001	68.8%	61.0%	65.4%	76.6%	0.0003
Mushrooms	36.5%	45.9%	53.3%	59.4%	<0.0001	41.3%	49.5%	53.2%	58.9%	<0.0001
Seaweeds	67.8%	55.1%	60.0%	63.8%	0.1	49.5%	46.7%	54.9%	60.2%	0.0008
Fish	73.0%	75.3%	82.4%	88.1%	0.0003	70.6%	69.0%	77.1%	85.4%	<0.0001
Meat	90.4%	97.3%	94.9%	93.0%	0.047	82.6%	91.6%	93.1%	91.9%	0.01
Eggs	73.9%	75.4%	80.3%	84.4%	<0.0001	77.1%	68.3%	76.6%	80.6%	<0.0001
Dairy	63.5%	60.1%	67.2%	76.5%	0.0002	78.0%	73.4%	77.4%	79.8%	0.37
Fats and oils	88.7%	91.6%	92.5%	91.3%	0.003	86.2%	87.1%	91.0%	90.3%	0.03
Confectioneries	31.3%	34.3%	35.1%	45.3%	0.29	55.0%	52.9%	53.2%	50.1%	0.45
Beverages	96.5%	95.9%	95.4%	96.3%	0.67	94.5%	94.6%	95.5%	96.9%	0.09
Condiments	98.3%	100.0%	100.0%	100.0%	na	98.2%	100.0%	100.0%	100.0%	na

NHNSJ, the National Health and Nutrition Survey in Japan; na, not applicable. * Crude values are shown in the table. “Shared meal occasion” means ≥1 food item (not included food groups of “sugars”, “fats and oils”, “beverages”, and “condiments”) recorded under breakfast, lunch, or dinner for a participant that was also consumed by ≥1 household member based on the application of the approximated proportion method. Results were calculated for participants with consumption ≥0 g/d. † Wald Chi-square test based on multivariate logistic regression adjusted for age groups (i.e., 20–29, 30–39, 40–49, 50–59, 60–69, 70–79, and ≥80 y), occupation (i.e., office/service, manual, agricultural related, and housework/other), meal skipping (i.e., yes or no), snack (i.e., yes or no), and characteristics of residence area (i.e., government-designated cities, population ≥150,000, 50,000–150,000, and <50,000, and town/village). Not applicable for small expected frequencies.

**Table 4:** Intake of food groups by household shared meal frequency in Japanese adults ≥20 years living with ≥1 household member from the 2018 and 2019 NHNSJs (n = 7196) *.

	Men (n 3310)											Women (n 3886)										
		0 Times/Day		1 Time/Day		2 Times/Day		3 Times/Day					0 Times/Day		1 Time/Day		2 Times/Day		3 Times/Day			
Food Groups (g/1000 kcal)	N (%) of Consumers	Mean	SE	Mean	SE	Mean	SE	Mean	SE	*p* for ANOVA	*p* for Trend †	N (%) of Consumers	Mean	SE	Mean	SE	Mean	SE	Mean	SE	*p* for ANOVA	*p* for Trend †
Cereals	3309 (100)	251.5	7.0	246.1	3.8	245.4	3.4	237.1	3.5	0.01	0.04	3875 (99.7)	228.7	7.2	219.0	4.1	218.7	3.6	218.7	3.7	0.52	0.14
Potatoes	2398 (72.4)	28.9	3.7	37.0	2.0	37.1	1.8	40.3	1.9	0.01	0.002	2739 (70.5)	35.7	4.6	47.5	2.5	44.1	2.2	47.0	2.3	0.01	0.02
Sugars	2619 (79.1)	4.7	0.5	4.5	0.3	4.5	0.2	4.8	0.2	0.22	0.87	3008 (77.4)	5.9	0.6	5.3	0.3	4.8	0.3	4.8	0.3	0.09	0.04
Pulses	2529 (76.4)	30.5	5.0	39.5	2.5	41.0	2.1	42.5	2.2	0.07	0.01	3028 (77.9)	49.5	6.4	56.8	3.5	55.0	3.1	55.4	3.2	0.70	0.38
Nuts	991 (29.9)	4.5	2.2	5.0	0.9	3.5	0.8	2.6	0.8	0.02	0.25	1221 (31.4)	3.7	1.4	6.6	0.9	4.3	0.8	4.1	0.8	0.006	0.77
Vegetables	3308 (99.9)	102.0	7.6	117.1	4.1	134.9	3.7	148.4	3.8	<0.0001	<0.0001	3877 (99.8)	127.5	9.3	141.9	5.2	153.6	4.6	167.7	4.7	<0.0001	<0.0001
Fruits	1995 (60.3)	67.5	9.1	56.2	5.2	57.7	4.5	61.5	4.6	0.3	0.5	2719 (70.0)	98.4	8.5	83.1	5.4	81.2	4.8	82.7	4.9	0.18	0.04
Mushrooms	1786 (54.0)	12.4	2.6	16.0	1.2	16.1	1.0	17.7	1.1	0.046	0.03	2135 (54.9)	8.9	2.6	21.3	1.2	20.7	1.1	21.6	1.1	<0.0001	<0.0001
Seaweeds	2020 (61.0)	5.8	1.3	8.5	0.8	8.6	0.7	7.4	0.7	0.04	0.19	2177 (56.0)	4.2	2.0	9.5	1.2	8.8	1.0	8.2	1.0	0.053	0.045
Fish	2754 (83.2)	44.4	3.7	45.1	2.0	46.4	1.8	44.2	1.8	0.49	0.94	3092 (79.6)	40.8	4.0	48.0	2.3	48.3	2.0	48.1	2.1	0.27	0.06
Meat	3123 (94.4)	61.0	3.5	61.7	1.9	61.8	1.7	62.8	1.8	0.86	0.60	3578 (92.1)	60.2	3.8	63.2	2.1	62.9	1.9	63.6	1.9	0.79	0.37
Egg	2680 (81.0)	22.6	2.0	24.6	1.1	26.6	1.0	29.1	1.0	<0.0001	0.0003	3004 (77.3)	32.6	2.4	30.4	1.4	29.0	1.2	29.8	1.2	0.28	0.16
Dairy	2309 (69.8)	72.9	8.0	66.5	4.6	59.5	4.1	59.9	4.2	0.12	0.049	3029 (77.9)	100.1	8.9	76.6	5.6	76.4	5.0	75.5	5.1	0.03	0.003
Fats and oils	3036 (91.7)	7.0	0.5	5.9	0.3	5.9	0.2	5.6	0.2	0.02	0.003	3498 (90.0)	7.0	0.5	6.8	0.3	6.4	0.3	6.3	0.3	0.07	0.06
Confectioneries	1295 (39.1)	41.1	3.8	33.1	2.2	31.9	2.0	29.4	2.0	0.011	0.002	2014 (51.8)	46.3	3.8	44.0	2.3	40.5	2.1	37.2	2.1	0.001	0.005
Beverages	3175 (95.9)	383.0	23.5	395.3	13.0	363.0	11.9	338.1	12.2	0.0001	0.02	3730 (96.0)	395.6	26.0	377.8	15.2	354.8	13.3	343.5	13.6	0.02	0.02
Condiments	3308 (99.9)	26.8	2.0	31.6	1.1	32.6	1.0	33.1	1.0	0.01	0.001	3884 (99.9)	27.3	2.3	33.1	1.3	34.0	1.1	34.7	1.2	0.006	0.0004

NHNSJ, National Health and Nutrition Survey in Japan; SE, standard error. * Shared meal refers to a meal occasion (i.e., breakfast, lunch, and dinner) with ≥1 food item (not included food groups of ‘sugars’, ‘fats and oils’, ‘beverages’, and ‘condiments’) that was consumed by ≥1 household member based on the application of the approximated shared proportion. The results were calculated for consumers (>0 g/day). Mean intake values were adjusted based on multilevel linear regression, including age groups (i.e., 20–29, 30–39, 40–49, 50–59, 60–69, 70–79, and ≥80 years), occupation (i.e., office/service, manual, agriculture-related, and housework/other), meal skipping (yes or no), snacking (yes or no), and characteristics of residence area (i.e., government-designated cities, population ≥150,000, 50,000–150,000, and <50,000, and town/village) as fixed effects and households as a random effect. † Based on linear contrasts across frequencies of shared meal occasions.

## Data Availability

The data presented in this study are available on request from the corresponding author. The data are not publicly available due to privacy and ethical concerns.

## Supplementary Materials

The following supporting information can be downloaded at: https://www.mdpi.com/article/10.3390/nu16111764/s1, Table S1. Basic characteristics of included and excluded participants.
