# Supplementary material for: Relationship between Household Shared Meal Frequency and Dietary Intake among Men and Women Aged ≥20 Years: Cross-Sectional Analyses Based on 2018 and 2019 National Health and Nutrition Surveys in Japan
Source: Nutrients. 2024 Jun 4;16(11):1764. doi: 10.3390/nu16111764 (PMC11175063; doi:10.3390/nu16111764)
Supplement: Supplementary file 1 [file nutrients-16-01764-s001.zip › nutrients-3025561-supplementary.pdf]

Table S1. Basic characteristics of included and excluded participants.

	Included Participants (n 7196)		Excluded Participants (n 2212)*		*p* †
	Mean	SD	Mean	SD	
Number of consumed food items	28.2	10.0	25.2	11.0	<.0001
Proportion of shared food times	0.66	0.25	0.66	0.28	0.99
Number of shared meal occasions	2.22	0.82	2.09	0.88	<.0001
EI (kcal)	1951	508.3	1818	729.8	<.0001
	N	%	N	%	
Gender					0.0004
Men	3310	46.0	1113	50.3	
Women	3886	54.0	1099	49.7	
Age group (years)					<.0001
20–29	456	6.3	271	12.3	
30–39	787	10.9	304	13.7	
40–49	1184	16.5	394	17.8	
50–59	1164	16.2	358	16.2	
60–69	1585	22.0	331	15.0	
70–79	1444	20.1	303	13.7	
≥80	576	8.0	251	11.3	
Occupation‡					0.0002
Office/service	3080	42.8	1007	45.5	
Manual	907	12.6	346	15.6	
Agricultural related	366	5.1	75	3.4	
Housework/other	2843	39.5	784	35.4	
Household size (persons)§					<.0001
2	2950	41.0	740	33.5	
3	1953	27.1	645	29.2	
4	1339	18.6	442	20.0	
≥5	954	13.3	385	17.4	
Meal skipping||					<.0001
Yes	341	4.7	317	14.3	
No	6855	95.3	1895	85.7	
Snacking¶					<.0001
Yes	4069	56.5	968	43.8	
No	3127	43.5	1244	56.2	
Characteristics of residence area					0.46
Government designated cities	1295	18.0	402	18.2	
≥150,000	2333	32.4	667	30.2	
50,000–150,000	2243	31.2	701	31.7	
<50,000	460	6.4	205	9.3	
Town/village	865	12.0	237	10.7	

EI, energy intake; NHNSJ, National Health and Nutrition Survey in Japan; SD, standard deviation.
* Excluded participants were those without records of height or weight (n 1818), underreporters (n 293), or overreporters (n 101) assessed based Goldberg's cutoff for a 1-day dietary record: 0.87–2.75.
† T-test for continuous variable; Mantel-Haenszel $\chi 2$ test for categorical variables.
‡ "Office/service" included professional, managerial, clerical, sales, and service workers; "manual" included security workers, transportation and machinery operators, and production process workers; "agricultural-related" included agricultural, forestry, and fishery workers; "housework/other" included housework, other, and students.
§ Based on the household member aged ≥1 year and participated ≥1 sub-survey consisted of the NHNSJ (i.e., the nutrition survey, the physical examination survey, and the lifestyle questionnaire survey).
| | "Yes" is defined based on meal types that were classified by participants as "dietary supplements only" or "no consumption" for breakfast, lunch, and dinner. "No" is defined based on meal types that were classified by participants as “home-cooked,” “take-out,” “dining out,” “provided-meal by school or nursery facility,” “provided-meal by work facility," or “sweets/fruits/dairy/beverages only.”
¶ Determined by if any food or beverage was recorded under the snack section in the dietary record booklet.
